# Targeting immunosuppression by TGF-β1 for cancer immunotherapy

**DOI:** 10.1016/j.bcp.2021.114697

**Published:** 2021-10

**Authors:** Grégoire de Streel, Sophie Lucas

**Affiliations:** de Duve Institute, Université catholique de Louvain, 1200 Brussels, Belgium

**Keywords:** Cancer immunotherapy, Tumor immunology, Regulatory T cells, TGF-β, GARP, Therapeutic monoclonal antibodies, Resistance to PD1 blockade

## Abstract

The TGF-β1 cytokine is a key mediator of many biological processes. Complex regulatory mechanisms are in place that allow one single molecule to exert so many distinct indispensable activities. The complexity of TGF-β1 biology is further illustrated by the opposing dual roles it plays during cancer progression. Risks of toxicities combined with lack of convincing therapeutical efficacy explain at least in part why therapies targeting TGF-β1 have lagged behind in past decades. However, recent successes of immunostimulatory antibodies for the immunotherapy of cancer and findings that TGF-β1 activity associates with resistance to immunotherapeutic drugs have revived the field. In this review, we discuss the biology of TGF-β1 with a special focus on its roles in regulating immune responses in the context of cancer. We describe the various therapeutic approaches available to inhibit TGF-β signalling, and more recent findings that allow selective targeting of specific sources of TGF-β activity, which may prove relevant to increase the efficacy and reduce the toxicity of cancer immunotherapy.

## Graphical abstract

**Figure:**
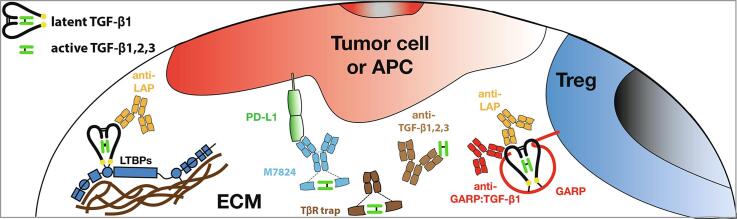

## 1 Introduction

The TGF-β superfamily comprises 32 ligands grouped in 2 subfamilies of TGF-βs and Bone Morphogenic Proteins (BMPs) [1]. The TGF-β subfamily comprises the three TGF-β1, -β2 and -β3, activins A and B, Nodal and several Growth and Differentiation Factors (GDFs). TGF-β1, -β2 and -β3 are encoded by 3 distinct genes and share 71–79% of amino-acid sequence identity in the C-terminal portion corresponding to the mature cytokine. Among the three TGF-βs, TGF-β1 is the most abundantly and widely expressed among immune cells, is the most abundant in serum and is most often the predominant isoform in the tumor microenvironment (TME). TGF-β1 is a key regulator of many biological processes including cell and tissue differentiation, vasculogenesis, wound healing and immune homeostasis. The importance of TGF-β1 in regulating immunity and inflammation is highlighted by the phenotype of *Tgfb1*^-/-^ mice, which die early after birth (3–4 weeks) from multifocal inflammation and auto-immune destruction of multiple organs [2].

Because it regulates a wide range of biological process, TGF-β1 production and activity are tightly controlled, mostly at post-translational levels, to control the biodisponibility of the cytokine. Further levels of regulation occur downstream of TGF-β1 binding to TGF-β receptors, via partnering of various transcription factors or regulators that ensure the specificity of TGF-β1 signaling in a given cellular context. Altogether, these mechanisms allow TGF-β1 to exert pleiotropic activities while preserving high specificity.

In this review, we will discuss the main aspects of TGF-β1 synthesis, activation and signaling, then focus on consequences of TGF-β1 signaling in the context of cancer. Finally, we will discuss strategies that allow to target TGF-β1 for the purpose of cancer immunotherapy.

### 1.1 Production of, and signaling by TGF-β1

#### 1.1.1 TGF-β1 synthesis

**Fig. 1:**
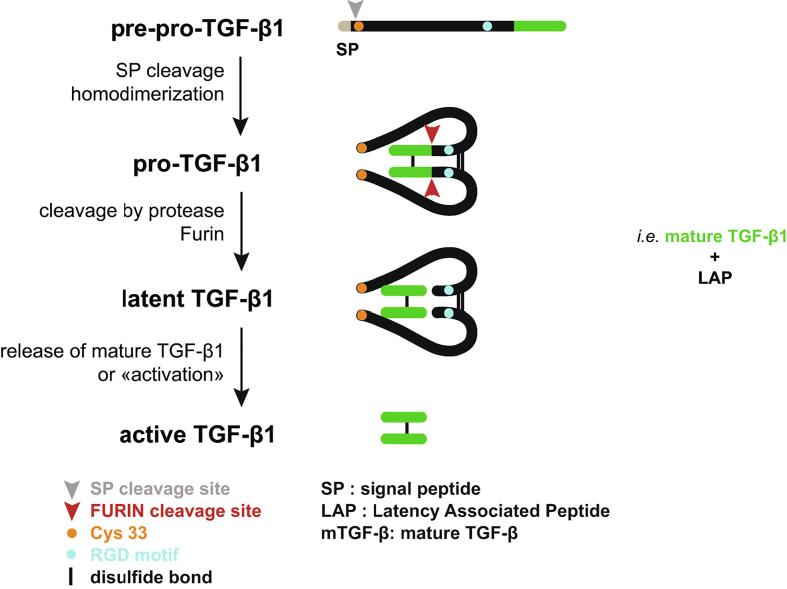
Schematic representation of TGF-β1 processing *(From Stockis et al, Mol. Biosystems, 2017).* TGF-β1 is first produced as a pre-pro-precursor. Following cleavage of signal peptide and homodimerization via formation of disulfide bonds, pro-TGF-β1 is cleaved by the pro-protein convertase Furin. The N-terminal dimer or Latency Associated Peptide (LAP), remains non-covalently associated with the C-terminal dimer or mature TGF-β1, to form an inactive complex called latent TGF-β1. To bind to its receptor, mature TGF-β1 must be released from LAP, a process called “TGF-β1 activation”.

All three TGF-βs are produced as pre-pro-TGF-β precursors that are addressed to the endoplasmic reticulum via a N-terminal signal peptide. After cleavage of the signal peptide, the pro-TGF-β precursors homodimerize by formation of three interchain disulfide bonds. They are then cleaved by the pro-protein convertase furin into a C-terminal portion called mature TGF-β, and a N-terminal latency associated peptide (LAP) which embraces the mature TGF-β. This non-covalent interaction prevents mature TGF-β from binding the TGF-β receptor. The mature TGF-β1:LAP complex is thus called “latent TGF-β1” (Fig. 1).

### 1.2 Reservoirs of latent TGF-β1

Latent TGF-β1 is produced by almost all cells of the organism, but most abundantly by hematopoietic and immune cells. It is either produced in the extracellular milieu as a soluble molecule, or it is tethered to other proteins via formation of disulfide bonds with cysteine 33 in LAP. Binding of TGF-β1 to one of the known latent TGF-β1 binding proteins excludes binding to the others, as they all form disulfide bonds with the same cysteine in LAP. Known latent TGF-β1 binding proteins comprise LTBP-1, −3 and −4, GARP and LRRC33. LTBPs do not possess a transmembrane domain. They tether latent-TGF-β1 in the extracellular matrix (ECM), by binding to fibronectin and fibrillins [3]. GARP, also called LRRC32, is a leucine rich repeat- containing protein. It has a large extracellular domain, followed by a transmembrane domain and a short cytoplasmic tail. Binding of latent TGF-β1 to GARP results in presentation of the inactive cytokine on the cell surface. It could also be involved in deposition of latent TGF-β1 in the ECM through shedding of the extracellular portion of GARP by an unidentified protease [4]. Expression of GARP:(latent)TGF-β1 complexes is restricted to a few cell types which include TCR-stimulated Tregs [5], [6], [7], BCR-stimulated B cells [8], fibroblasts, endothelial cells [9], megakaryocytes and platelets [10], mesenchymal stromal cells [11] and hepatic stellate cells [12]. LRRC33, or NRROS, is another leucine rich repeat- containing protein that shares moderate amino-acid sequence identities with GARP (34%). LRCC33 has a large extracellular domain and a short cytoplasmic tail and is mainly expressed on macrophages and microglia cells [13].

Altogether, disulfide linkage of latent TGF-β1 to large TGF-β1 binding proteins results in accumulation or storage of the inactive cytokine in the ECM, or at the surface of various cell types.

### 1.3 Activation of latent TGF-β1

TGF-β1 activation is a process by which the mature TGF-β1 dimer is released from the LAP to allow binding to the TGF-β receptor (Fig. 1). Whereas almost all cells produce latent TGF-β1, only a few were described to activate it. Activation is a critical step in TGF-β1 biology. Mechanisms by which TGF-β1 is activated vary depending on the specific pool of latent TGF-β1 that is involved in a given context.

Mechanisms of TGF-β1 activation that were proven important *in vivo* imply either Thrombospondin 1 or RGD-binding integrins. A KRKF motif in Thrombospondin 1 binds an LSKL motif in LAP. Mice homozygous for an inactivating mutation of Thrombospondin 1 present some of the symptoms of *Tgfb1^-/-^* mice, including multi-organ inflammation. But their symptoms are not as severe as *Tgfb1^-/-^* mice, suggesting the existence of additional important mechanisms of TGF-β1 activation *in vivo* [3].

A subset of the ~ 30 known mammalian integrins bind an RGD motif in their ligand. Integrins αVβ1, αVβ6 and αVβ8 bind the RGD motif present in the C-terminal domain of LAP from TGF- β1 and TGF- β3, but not from TGF- β2. These integrins are implicated in TGF-β1 activation *in vivo*, as illustrated in mice carrying a knock-in mutation of the *Tgfb1* gene which changes the RGD motif in LAP into RGE. This mutation abolishes integrin binding and recapitulates all symptoms observed in *Tgfb1^-/-^* mice [14].

Integrin αVβ1 is expressed by fibroblasts and appears to play roles in fibrosis development in lung and liver [15].

Expression of integrin αVβ6 is restricted to epithelial cells. Mice deficient in αVβ6 exhibit exacerbated lung and skin inflammation in response to minor insults, and are protected from skin and lung fibrosis [3]. Activation of latent TGF-β1 by αVβ6 involves contractile forces which require on one side polymerization of actin/myosin filaments located close to the cytosolic tail of the integrin, and on the other side, disulfide bond formation between LAP and LTBP immobilized in the ECM. These contractile forces result in deformation of the latent TGF-β1 complex and release of the mature cytokine [16]. The model involving contractile forces in latent TGF-β1 activation is supported by resolution of the latent TGF-β1 3D structure, which reveals how a latency lasso in the LAP hides binding sites of mature TGF-β1 to its receptor. These structural analyses show how unfolding of the latency lasso in LAP by linear forces applied by integrins to the opposite RGD binding site results in TGF-β1 activation. Disulfide anchorage of LAP to LTBP is required for unfolding of the LAP as a result of pulling by integrin αVβ6 [17].

Integrin αVβ8 is another RGD binding integrin that was described to activate latent TGF-β1. αVβ8 activates latent TGF-β1 from GARP:TGF-β1 complexes [18]. The β8 chain is expressed by murine DCs, human monocytes, neurons, astrocytes, airway epithelial cell, fibroblast, tumor cells and Tregs. In Tregs, TCR stimulation increases expression of the *Itgb8* mRNA by 8 to 10-fold. In contrast, the *Itgb6* mRNA remains undetectable in activated Tregs, and *Itgb1* is expressed at comparable levels by activated Tregs and non-regulatory T cells, although the latter T cells do not activate latent TGF- β 1 [18], [19], [20]. Thus, αVβ8 appears to be the only αV-containing RGD-binding integrin that contributes to latent TGF- β1 activation by Tregs. To the best of our knowledge, no other mechanism of latent TGF-β activation by Tregs has been described thus far.

**Fig. 2:**
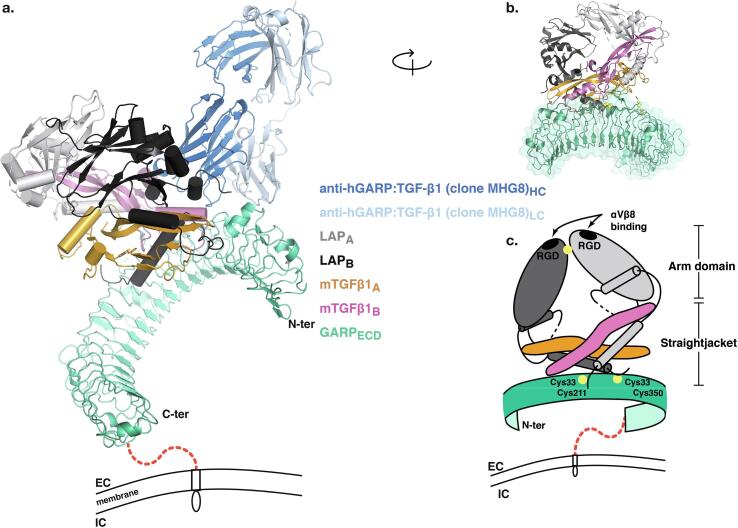
Overall structure of the GARP_ECD_:TGF-β1:MHG-8 Fab complex *(Figure from Liénart et al., Science, 2018).* a Cartoon representations of the tridimensionnal structure of GARP_ECD_:TGF-β1:MHG-8Fab complexes , as determined by X-ray crystallography. MHG-8 : anti-human GARP:TGF-β1 mAb ; ECD : extracellular domain. Latent TGF-β1 is composed of two LAP monomers (LAP_A_ and LAP_B_) and two mature TGF-β1 monomers (mTGF-β1_A_ and mTGF-β1_B_). Red dashed line: region located between the the transmembrane domain and the large extracellular, leucine-rich-repeat containing domain of GARP (juxta-membrane region not present or solved in the crystals). b Cartoon representation of a different view of the GARP:TGF-β1 complex without the MHG-8 Fab, not illustrated for clarity. c Schematic representation of protein components participating in the GARP_ECD_:TGF-β1 complex. (For interpretation of the references to colour in this figure legend, the reader is referred to the web version of this article.)

Strikingly, *Itgb8^-/-^* mice die during embryonic development or immediately after birth of vascular defects in the central nervous system. In addition, *Itgb8^-/-^* mice harbor cleft palate, a phenotype similar to that of *Tgfb3^-/-^* mice [3]. In contrast, mice that lack β8 specifically in Tregs develop normally, and do not show signs of spontaneous inflammation [19], [21]. *Itgb8^-/-^* Tregs were capable of preventing colitis when they were co-transferred with naïve T cells to *Rag1^-/-^* mice. However, *Itgb8^-/-^* Tregs failed to control ongoing colitis when they were injected two weeks after the transfer of naïve T cells in *Rag1^-/-^* mice. This inability of *Itgb8^-/-^* Tregs to control established inflammation was not due to alteration in Treg maintenance, homing or survival, but to their inability to produce active TGF-β1 [21]. In human tumors, αVβ8 appears to be widely expressed by tumor cells and was suggested to contribute to activation of latent TGF-β1 presented by non-tumor cells [20]. The precise molecular mechanisms of TGF-β1 activation *via* αVβ8 is not completely understood. The sequence of the intracytoplasmic tail of the β8 chain is very different from that of β6. In contrast to αVβ6, deletion of the intracytoplasmic tail of β8 does not abrogate the ability of αVβ8 to activate TGF-β1 [22]. It is thus not clear whether contractile forces exerted by the cytoskeleton and transmitted by αVβ8 are at stake for TGF-β1 activation by αVβ8. Several authors report that recruitment of the MMP-14 protease by αVβ8 is required to induce degradation of the LAP and activate TGF-β1 in some cell types [22], [23]. However, proteases do not appear to be required for TGF-β1 activation by αVβ8 in Tregs [18]. αVβ8-mediated TGF-β1 activation from GARP:TGF-β1 complexes on Tregs requires disulfide linkage of latent TGF-β1 to GARP, and anchoring of GARP in the Treg membrane [4], [18]. Our lab developed antibodies that bind GARP:TGF-β1 complexes on Tregs and prevent αVβ8-mediated activation of TGF-β1 (Fig. 2). These anti-GARP:TGF-β1 antibodies do not prevent interaction of GARP:TGF-β1 complexes with αVβ8, but appear to prevent elongation of the LAP by αVβ8 [24], [25]. A recent analysis suggests indeed that binding of αVβ8 to LAP induces a structural deformation of the LAP unmasking mature TGF-β1 sites that are covered by the latency lasso [26]. This would allow the mature cytokine to access TGF-β receptors without actual release of a soluble form of the cytokine in the extracellular space. The authors confirmed the hypothesis of TGF-β1 activation in the absence of release of soluble mature TGF-β1 by using a TGF-β1 mutant that cannot be cleaved by furin (Fig. 1).

Altogether, Tregs appear to be able to activate latent TGF-β1 from GARP:TGF-β1 complexes on their surface thanks to tensile forces transmitted by αVβ8. This may not require actual release of soluble mature TGF-β1 in the extracellular medium, but rather a conformational change that allows access of the mature cytokine to TGF-β receptors on neighboring cell membranes. Whether αVβ8 needs to be present on the same Treg cell as GARP:TGF-β1 complexes is not known yet. The mature TGF-β1 exposed via this activation mechanisms can act in both a paracrine fashion on neighboring cells and an autocrine fashion on Tregs themselves.

### 1.4 Signaling by TGF-β1

**Fig. 3:**
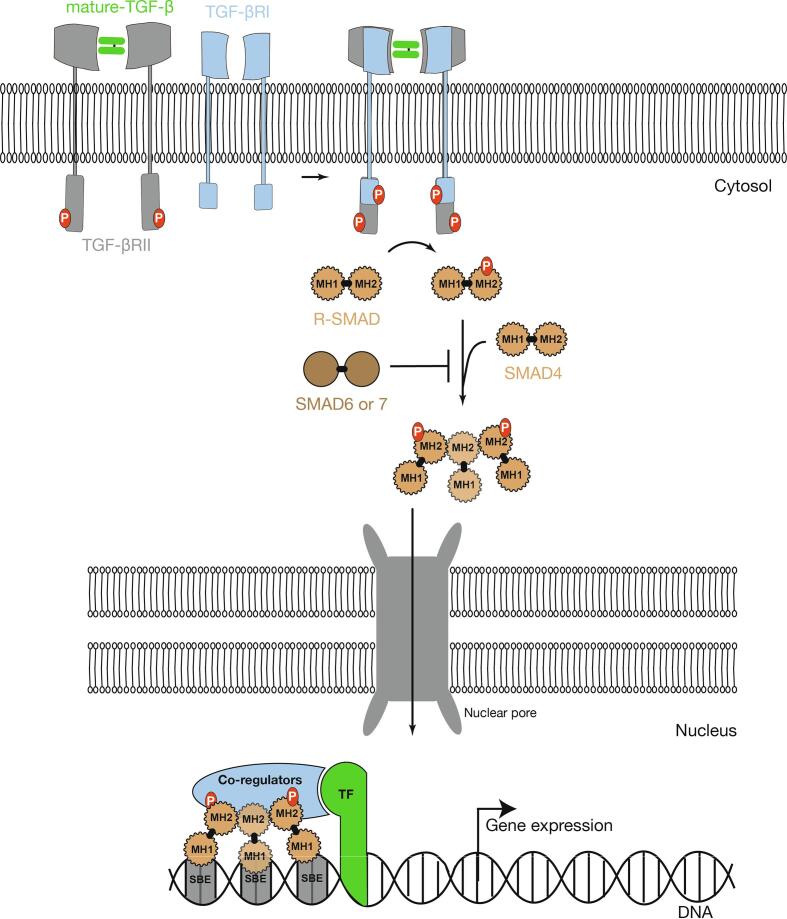
Canonical TGF-β signaling. Mature TGF-β first binds to TGF-βRII chains that in turn recruit TGF-βRI chains to complete the full heterotetrametric TGF-βR. TGF-βR contains serine/threonine kinase domains that phosphorylate the receptors-regulated SMADs (R-SMADs), SMAD2 or SMAD3. Once phosphorylated R-SMADs are able to form complexes with other R-SMADs and SMAD4 by association through MH-2 domains. The SMADs complexes translocate to the nucleus where, through MH1 domains, they can bind DNA on SMAD binding sites (SBE). SMADs associate with other Transcriptional Factors (TF) and co-regulators to regulate the expression of target genes.

TGF-β1, -β2 and -β3 signal through a hetero-tetrameric receptor bringing together two TGF-βRII chains and two TGF-βRI chains. TGF-βRI and II chains have serine-threonine kinase activity. The cytokine first binds to TGF-βRII chains, which recruit and phosphorylate intra-cytosolic domains of TGF-βRI chains. These in turn recruit and phosphorylate R-SMADs. R-SMADs (SMAD1, −2, −3, −5, −8) belong to the SMAD family, which comprises a total of eight members. SMAD2 and 3 are the main targets of TGF-βR kinase activity. Phosphorylation of serine residues on SMADs generate an acidic tail required for oligomerization between their MH2 domain and SMAD4. Then, SMAD dimers or trimers translocate to the nucleus, where they regulate expression of genes carrying SMAD-responsive regulatory regions. Even though SMAD complexes have some DNA binding activity via their MH1 domain, they need to associate with other DNA-binding cofactors to reach high binding affinity and target specific genes [27]. The diversity of SMAD cofactors also contributes to the pleiotropy of action of TGF-β cytokines (Fig. 3).

In addition to SMAD4, R-SMADs also associate with TIF1γ or IKKa proteins, which can also contribute to TGF-β signaling via the SMAD pathways [27]. Finally, TGF-β is also able to deliver signals via SMAD-independent pathways, which imply MAP kinases, PI3 kinases and Rho GTPases. Excellent reviews are available that describe SMAD and non-SMAD pathways transducing TGF-β signals and their roles in cancer [28], [29], [30], [31], [32], [33].

There are many levels of regulation of TGF-β signaling once the cytokine has bound its receptor. A few examples include protein FKBP1A, which locks TGF-βRI in an inactive conformation; SMAD7, which recruits the E3 ubiquitin-ligase SMURF2 to increase TGF-βRI and II turnover; SMAD6, which interferes with SMAD2 phosphorylation and interacts with SMAD4 to prevent heteromerization; or yet ZFYV9 and endofin, which regulate R-SMAD phosphorylation [1]. SMAD trafficking to the nucleus is also highly regulated by nuclear pore complex proteins (NUP153, NUP214), and R-SMAD phosphorylation is regulated by phosphatases PPM1A, PP2A in the nucleus inducing shuttling back to the cytoplasm.

SMAD-dependent and SMAD-independent signaling by TGF-β1 results in regulation of the expression of sets of genes. The sets of genes that are induced or repressed by TGF-β1 signals in a given cell type are called TGF-β1 signatures. TGF-β1 signatures are extremely diverse and context-dependent: different cell types respond to TGF-β1 by inducing and repressing largely non-overlapping gene sets. This is due mainly to SMADs partnering with a wide variety of transcription factors or co-factors specific of lineage differentiation (LDFs for Lineage Differentiation Transcription Factors) or induced by various other signals (SDFs for Signal Driven Transcription Factors (SDFs).

## 2 Roles of TGF-β1 in cancer

**Fig. 4:**
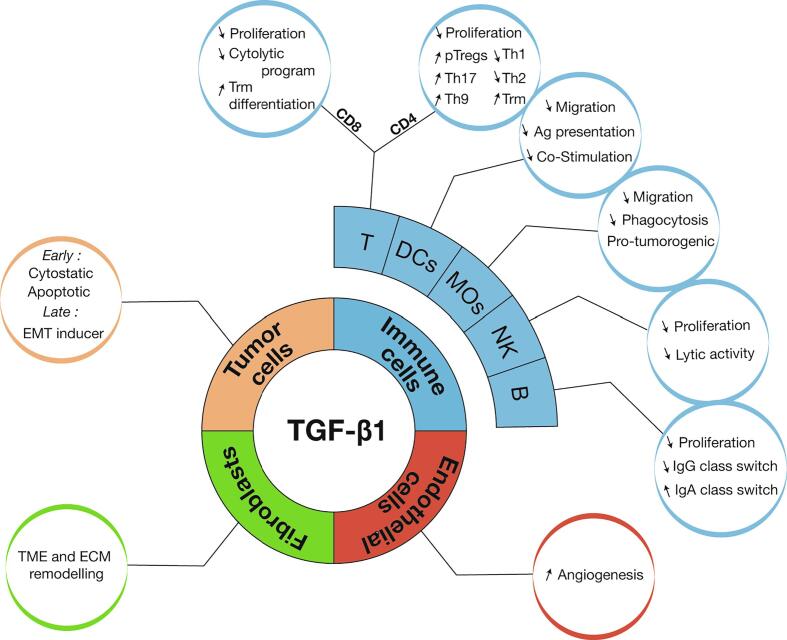
TGF-β1 activities in cancer. TGF-β1 exerts multiple activities in cancer. In pre-malignant and early malignant lesions, TGF-β1 exerts anti-tumor activity, but switches to pro-tumorigenic functions in established tumors by favoring EMT, inhibiting proliferation, differentiation and anti-tumor functions of multiple immune cells. In addition, TGF-β1 favors the establishment of an immunosuppressive tumor microenvironment through modulation of immune and stromal cell functions.

TGF-β1 exerts a vast array of activities during embryonic development, cell and tissue differentiation, vasculogenesis, wound healing, immune response and tumorigenesis. Here, we will discuss exclusively the activities that it exerts in the context of cancer (Fig. 4). We will briefly summarize how it acts on tumor cells themselves and on cells that infiltrate tumor lesions, with a specific emphasis on the regulation of anti-tumor immune responses. A more detailed review of TGF-β biology in cancer progression and consequences for cancer immunotherapy can be found here: [34].

### 2.1 Activities of TGF-β1 on tumor cells

The roles of TGF-β1 signals in tumor cells are often described as “dual”: TGF-β1 exerts potent cytostatic and pro-apoptotic activities in pre-malignant cells, but also favors EMT and metastases at later stages of malignant transformation. Cytostatic effects on epithelial and hematopoietic cells are due to the fact that TGF-β1 induces cyclin-dependent kinase inhibitors (CDKis), represses c-Myc and inhibits translation by inducting the 4EBP1 protein [35].

TGF-β1 signals shift from tumor-suppression to tumor-promotion during progression of cancer. This is often associated with a partial loss of TGF-β signaling in tumor cells. For example, the anti-proliferative pressure exerted by TGF-β1 on pre-malignant cells selects cells with mutations in the TGF-β signaling pathway molecules. This, in turn, potentiates other oncogenic signals such as those of mutated Ras-MAPK pathways [36]. Mutations in TGF-β signaling molecules include for example biallelic inactivation of the *TGFBR2* gene in colon, gastric, biliary, pulmonary, ovarian, esophageal and head and neck carcinomas. They are particularly frequent in tumors with high microsatellite instability, due to a sequence in *TGFBR2* prone to replication errors [27]. Another example are mutations in the *SMAD4* gene, found in half of pancreatic ductal adenocarcinomas and microsatellite stable colorectal cancers [37], [38], [39]. Inactivation of TGF-βRII or inactivation of SMAD4 could protect transformed cells from the cytostatic and pro-apoptotic activities of TGF-β1, while maintaining SMAD-independent, pro-tumoral TGF-β1 signals in the case of SMAD4 inactivation [1].

However, a large proportion of tumors such as melanomas, gliomas, breast adenocarcinomas, have a perfectly functional TGF-β signaling pathway. In these cases, the cytostatic effects of TGF-β1 are often bypassed by mutations in other tumor suppressor pathways or activation of oncogenes.

When freed from the TGF-β cytostatic and pro-apoptotic activities, cancer cells exploit other facets of TGF-β signaling which favor tumor progression. This includes effects on other cells of the tumor microenvironment, which we will discuss later. But importantly, TGF-βs also induce so-called epithelial-to-mesenchymal transition (EMT) in malignant cells, a critical step for metastasis formation. When undergoing EMT, epithelial cells loose apicobasal polarity and downregulate key epithelial cell-adhesion molecules such as E-cadherin, which confers cell motility. In molecular terms, TGF-β signals induces SNAIL and ZEB1, two transcriptional repressors of E-cadherin expression, and TGF-βRII phosphorylates Par6, involved in the dissolution of cell junctions [1].

### 2.2 Activities of TGF-β1 on cancer associated fibroblasts (CAFs)

Even in tumors in which cancerous cells have completely lost TGF-β responsiveness, TGF-β1 can favor tumor progression by exerting effects on normal cells of the tumor microenvironment. Calon and colleagues, for example, demonstrated that CAFs able to respond to TGF-β1 signals are required to unable the implantation of transplanted colo-rectal carcinoma cells in mice [40]. CAFs are tumor promoting cells, deriving from resident tissue fibroblasts, adipose tissue stem cells, or others cells that have been exposed to TGF-β1. They produce high amounts of ECM proteins and proteases involved in the ECM remodeling, overall generating a favorable environment for tumor progression. CAFs secrete themselves high amounts of latent TGF-β1, although how and whether they actually activate the cytokine is not clear. They thus appear to contribute to a positive feedback loop, creating a niche favoring immunosuppression and EMT [41]. Navab et al showed that a CAF associated gene signature was associated with poor prognosis in NSCLC patients [42].

### 2.3 Activities of TGF-β1 on endothelial cells

TGF-β1 is involved in vasculogenesis during development. In addition, TGF-β1 is required for endothelial cell integrity as indicated by hemorrhagic syndromes observed in individuals bearing mutation that disturb TGF-β1 signaling [43]. In the context of cancer, TGF-β1 signaling sustains a pro-angiogenic environment notably through upregulation of VEGF pro-angiogenic factors [44]. Angiogenesis is an important component of tumor progression because it provides tumor cells with a source of oxygen and nutrients. Moreover, it favors EMT and dissemination of transformed, malignant cells during metastasis formation. TGF-β1 can be produced by endothelial cells themselves and could therefore suppress immune cells at their point of entry into the tumor site.

### 2.4 Production of TGF-β1 and its activities on tumor-infiltrating immune cells

#### 2.4.1 T cells

TGF-β signals exert major impacts on T cell development and functions [1], [45]. This is best illustrated by the phenotype of mice that have a conditional deletion of the *Tgfbr2* gene in T cells (*Cd4^cre^* × *Tgfbr2*^fl/fl^). These mice show the same multi-organ inflammation that is observed in *Tgfb1^-/-^* mice, and die 3–4 weeks after birth [46]. Of course, this phenotype precludes analysis of tumor development and progression in the absence of TGF-β1 signaling in T cells. In earlier work however, Gorelik and Flavell had generated transgenic mice expressing a dominant negative TGF-βRII in T cells. *Cd4-Tgfbr2^dn^* transgenic mice developed milder, delayed inflammation and autoimmunity by comparison to *Cd4^cre^* × *Tgfbr2*^fl/fl^ mice, due to incomplete abrogation of TGF-β signaling in T cells [47]. This allowed the authors to demonstrate that attenuation of TGF-β signaling in T cells renders mice resistant to tumor growth, using two different transplantable tumor models (EL4 thymoma and B16/F10 melanoma). Tumor-bearing transgenic mice developed more potent tumor-specific CTL responses than non-transgenic controls [48]. Interestingly, although increased anti-tumor immunity in transgenic mice required the presence of both CD8 and CD4 T cells, it required attenuation of TGF-β1 signals in CD8 T cells, but not in CD4 T cells. This was shown using adoptive transfers of CD4 and CD8 T cells expressing the dominant negative TGF-βRII in *Rag1^-/-^* mice bearing EL4 tumors. Using a similar transfer approach, the authors also showed that to exert anti-tumor activity, CTLs expressing the dominant negative receptor must be transferred early (3 days) after tumor cell implantation. If T cells were transferred at a later time point (day 7), ^dn^TGF-ßRII expressing CTLs did not exert anti-tumor activity. This suggests that TGF-β signals in CD8 T cells are immunosuppressive at early stages (induction) of anti-tumor immune responses. Another explanation could be that the tumoral burden at day 7 is too high to allow elimination of the tumor by CTLs. Later, Thomas and Massague generated EL4 cells expressing a short hairpin RNA (shRNA) targeting *Tgfb1* and showed that the cellular source of TGF-β1 blocking anti-tumor T cells is not tumor cells [49]. Others later suggested that the source could be T cells themselves. Indeed, in an oncogene-induced model of prostate cancer in mice, attenuation of TGF-β signalling in T cells resulting from expression of the *Cd4-Tgfbr2^dn^* transgene was associated with increased infiltration of tumors by CD4 and CD8 T cells expressing high levels of IFNγ and Gzmb [50]. The authors made similar observations in mice carrying a deletion of the *Tgfb1* gene in T cells (*Cd4*^cre^ × *Tgfb1*^fl/fl^ mice), but not in Tregs (*Foxp3*^cre^ × *Tgfb1*^fl/fl^ mice). This suggested that the source of the TGF-β1 that blocks anti-tumor immunity are T cells, but not Tregs. However, it does not rule out the possibility that Tregs may be implicated by activating the latent TGF-β1 produced by other T cells.

TGF-β1 is a potent inhibitor of T cell growth, partly because it inhibits IL-2 expression and secretion by T cells themselves [49]. In addition to inhibiting T cell proliferation, TGF-β1 signals directly target CTL effector programs, inhibiting expression of the *Ifng, Gzma*, *Gzmb*, *Prf1* and *Faslg* genes. Repression of *Ifnγ* and *Gzmb* by TGF-β1 is mediated by SMADs cooperating with ATF1 and CREB transcription factors [49]. Using *Cd4-Tgfbr2^dn^* mice, it was shown that TGF-β signals do not modify the expression of activation markers, such as CD69 and CD44 on CD8 tumor infiltrating T lymphocytes (TILs.) This suggests that TILs undergo proper activation but fail to induce their cytolytic effector program when they are under the influence of TGF-β1. Interestingly, IL-2 was sufficient to rescue the growth and effector functions of CD8 T cells exposed to TGF-β1, *in vitro*, indicating that the inhibitory activity of TGF-β1 on CD8 T cells is reversible. Much later, TGF-β1 was shown to induce PD-1 expression on human CD8 and CD4 T cells that were stimulated *in vitro*. This effect was dependent on SMAD3, but not SMAD2, and required interaction with NFATc1 to bind SMAD-binding elements (SBEs) on the *Pdcd1* promoter [51]. TGF-β1 influences T cell functions in many other ways which could be relevant to cancer, although this has not been formally demonstrated. For example, CD4 T cells exposed to TGF-β1 fail to differentiate into Th1 or Th2 cells because of direct downregulation of the transcription factors T-bet and GATA-3, respectively. TGF-β1 inhibits IFNγ production by already differentiated Th1 cells [52], [53]. In the presence of IL-6 or Il-4, TGF-β1 was shown to induce differentiation of CD4 T cells into Th17 or Th9 cells, respectively, but the role of these subsets of T helper cells in tumors is not clear [54]. In addition, TGF-β1, notably through its role in inducing expression of the CD103 integrin, influences the homing of T cells to tissues. TGF-β1 favors the establishment of tissue resident memory T cells (Trm), which play critical roles in preventing infections in epithelial tissues [55], [56].

TGF-β1 can also affect anti-tumor T cell responses by downregulating MHC molecules on the surface of tumor cells.

Finally, it is worth noting that although TGF-β1 is an immunosuppressive cytokine that inhibits establishment of potent anti-tumor immune responses, its anti-inflammatory activity could nevertheless also reduce tumor development. This may be relevant in mucosal tissues such as the gut, where colon inflammation is associated with a higher risk of colon cancer development in humans [27], [57].

#### 2.4.2 Tregs

Tregs are a subset of immunosuppressive CD4 T cells that are required for the maintenance of immune tolerance in the periphery. They are characterized by expression of transcription factor FOXP3. Two main subsets of Tregs can be distinguished based on the anatomical site where they acquire their Treg identity. Some T cell precursors differentiate into tTregs in the thymus (hence their name), whereas some naïve CD4 T cells differentiate into pTregs upon antigen encounter in the periphery. Although this may not be as simple as this, TGF-β signals appear not to be required for tTreg lineage commitment in the thymus but to be required for pTreg induction and maintenance in the periphery [46]. TGF-β1 induces pTregs differentiation thanks to SMAD and NFAT, which induce *FOXP3* expression in naïve CD4 T cells. pTregs appear to play critical roles in suppressing T cell responses against harmless commensal antigens [58]. It is interesting to mention that mice lacking TGF-β1 specifically in Tregs (*Foxp3^cre^ × Tgfb1^fl/n^*) have increased numbers of Tregs in the lymph nodes, indicating that autocrine TGF-β1 might be implicated in the control of homeostatic, and perhaps also antigen-induced proliferation of Tregs [59].

Tregs are known to play deleterious roles in multiple human cancers [60], [61], [62], [63], [64]. It is important to note that it is very difficult, if not impossible at the present time, to distinguish tTregs from pTregs in humans. No good marker is yet available. For that matter, FOXP3 expression itself is not a specific marker of human Tregs either, as many non-regulatory T cells induce transient FOXP3 expression in response to stimulation. Our lab and others have shown that human and mouse Tregs, but not other T cells, produce and activate TGF-β1 upon stimulation of their TCR [65]. We proposed that TGF-β1 production is a key mechanism by which Tregs supress other immune cells. We showed *in vitro*, using human Treg clones or polyclonal Tregs, that production of active TGF-β1 by Tregs requires GARP and integrin αVβ8 [18], [24], [25]. Work by others supports a role for Treg-derived TGF-β1 in suppressing anti-tumor immunity *in vitro*. For example, anti-HA Tregs were shown to abrogate the anti-tumor activity of anti-HA CD8 T cells against HA-expressing CT26 tumors in a TGF-β dependent manner [66]. In this model, Tregs did not inhibit the proliferation, differentiation or cytokine production by anti-HA CD8 T cells, but only reduced their cytolytic activity.

We recently showed that GARP-expressing Tregs were required for the anti-tumor activity of antibodies that block production of active TGF-β1 from GARP:TGF-β1 complexes on the surface of Tregs in mice transplanted with syngeneic colon carcinoma cells [67].

#### 2.4.3 Dendritic cells

Dendritic cells (DCs) are a subset of myeloid cells specialized in antigen processing and presentation to T cells. They are required for the proper priming and development of T cell responses. TGF-β1 prevents the maturation of DCs, reducing antigen presentation and expression of co-stimulatory molecules [52]. TGF-β1 also affects DC migration to the lymph nodes, and inhibits expression of MHCII molecules [68], [69]. DCs isolated from tumors of mice lacking TGF-βRII in the myeloid compartment had an increased ability to induce anti-tumor T cell proliferation *in vitro*, by comparison to DCs from WT mice [70].

TGF-β1 exerts potent effects on many other immune cell types, including macrophages, NK cells and B cells (Fig. 4). Notably TGF-β1 represses expression of the activating receptor NKG2D on NK cells. However, the consequences of TGF-β1 signaling in these other immune cells in the context of cancer has not yet been thoroughly explored and elucidated, due in part to the lack of appropriate tools and models. Notwithstanding this, TGF-β1 is seen as a very interesting target for the purpose of cancer immunotherapy.

## 3 Targeting TGF-β1 for the immunotherapy of cancer

**Fig. 5:**
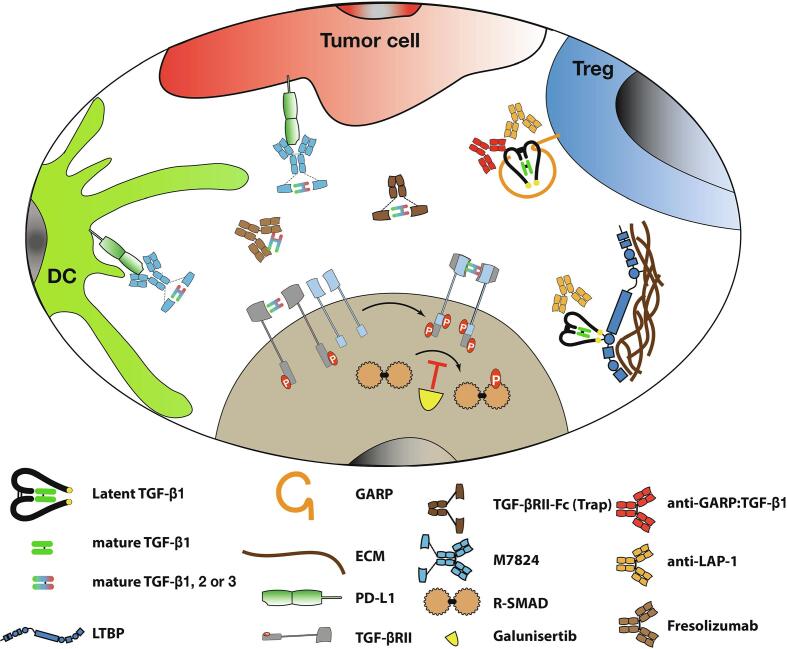
Strategies to target TGF-β in cancer. Multiple strategies have been developed to target TGF-β activity. They differ in their specificity, particularly with regards to the reservoir of latent TGF-β that is targeted. Galunisertib is a TGF-βRI kinase inhibitor that prevents phosphorylation of R-SMADs. Fresolizumab and TGF-βRII-Fc (Trap) neutralize free, mature TGF-β1, -β2, -β3 isoforms. Bifunctional molecules such as M7824 capture free mature TGF-β1, -β2, -β3 isoforms in close vicinity with PD-L1 expressing cells. Anti-LAP and anti-GARP:TGF-β1 mAbs are specific of TGF-β1 (*vs* TGF-β2 or TGF-β3) and block latent TGF-β1 activation. Anti-LAP mAbs block activation of all reservoirs of latent TGF-β1, whether it is bound to LTBPs in the extracellular matrix, to GARP on the surface of Tregs and platelets, or to LRRC33 on the surface of myeloid cells (not shown). Anti-GARP:TGF-β1 block only the activation of latent TGF-β1 presented by GARP on the surface of GARP-expressing cells (*i.e.* mostly Tregs and platelets).

Owing to its roles favoring cancer progression and metastases and suppressing anti-tumor immunity, TGF-β1 has remained a coveted target for the purpose of cancer therapy. A wealth of clinical reports associate high TGF-β1 levels with poor prognosis in many cancer types [71], [72], [73], [74], [75]. Nonetheless, progress in the development of TGF-β1-targeting therapies have been slow, probably because of the fear of severe toxicities that could arise from blocking tumor suppression exerted by TGF-β1 at early stages of tumorigenesis [57]. Toxicities could also occur as a result of blocking TGF-β1 activities on normal cells outside of the tumor microenvironment. Despite these concerns, multiple strategies are under development to block TGF-β1 in cancer, and some have reached clinical trials (Fig. 5).

### 3.1 Inhibitors of TGF-β receptor activity

Galunisertib (LY2157299 monohydrate) is the most advanced TGF-βRI kinase inhibitor in the clinics. Like all other TGF-βRI inhibitors, it has raised concerns because of cardiac toxicities [76]. TGF-βRI was shown to be essential for heart homeostasis in adult rats, ensuring proper tissue repair of heart valves exposed to strong mechanical stress [77]. Compared to other TGF-βRI inhibitors, galunisertib made its path to the clinic thanks to its lower toxicity in animals, which appeared only at high doses or upon continuous administration during several months. A considerable effort was made in order to adapt dosage, to meet both criteria of safety and efficacy in humans. Administration twice a day was required because of the very short half-live of the compound. Intermittent dosing twice a day during 14 days, followed by 14 days off treatment, was evaluated as sufficiently safe to avoid cardiac toxicities and chronic inflammation [76]. A first phase I dose escalation trial in glioblastoma patients revealed 16% of objective responses with no serious treatment-related toxicities, and in particular, no cardiac toxicities [78]. A subsequent phase II trial in glioblastoma patients compared treatment with chemotherapy (lomustine), galunisertib, and a combination of both. It revealed no increase in Overall Survival (OS) or Progression Free Survival (PFS) compared to chemotherapy [79]. By comparison to the phase I study, patients in the phase II study appeared to have disease progressing more rapidly. This may have accounted for the lack of efficacy of galunisertib observed in the phase II trial. In addition, combination of galunisertib with concomitant chemotherapy could have been counter-productive if galunisertib-stimulated immune cells were in turn depleted by chemotherapy [80]. In a more recent phase II trial, in pancreatic cancer patients, combination of chemotherapy (gemcitabine) and galunisertib showed increased survival over chemotherapy alone [81]. Based on several pre-clinical studies, galunisertib is now tested in clinical trials in combination with anti-PD-1 antibodies. A new TGF-βRI kinase inhibitor, vactosertib, has emerged as a more potent and specific molecule, and is also currently tested in early stage clinical trials [57].

None of the inhibitors of the kinase activity of TGF-βRI are expected to exert selective activity on tumor cells or immune cells within tumors. In addition, none inhibit TGF-β1 signals specifically, as TGF-β2 and TGF-β3 signal via the same receptor as TGF-β1.

### 3.2 Inhibitors that prevent binding of mature TGF-β cytokines to their receptor

#### 3.2.1 Neutralizing antibodies against the mature TGF-β cytokines

Fresolizumab is a fully human monoclonal IgG4 antibody that neutralizes mature TGF-β1, -β2 and -β3. Twenty-eight melanoma and one renal cell carcinoma patients received the antibody in a phase I dose escalation study. Forty-five percent of patients experienced at least one serious adverse event, with drug-related skin lesions being the most recurrent. These lesions comprised squamous cell carcinoma, hyperkeratosis and eruptive keratoacanthomas. All regressed after treatment completion. No grade IV or V adverse event, and no drug-related death occurred [82]. This safety profile was deemed manageable. A phase II trial was launched in patients with malignant pleural mesothelioma, but during the enrollment phase, testing of fresolizumab for oncology indications was interrupted by the manufacturer. Nonetheless, 13 patients had been recruited, but no objective response was reported. One patient developed a squamous cell carcinoma, and another experienced an accelerated progression of tumor under treatment [83].

#### 3.2.2 Antibodies against TGF-βRII

LY3022859, a human IgG1 anti-TGF-βRII monoclonal antibody was recently tested in a phase I clinical trial in 14 patients with diverse cancer types. Infusion-related toxicities were reported in the highest dose cohort (1.25 mg/kg), which was consequently discontinued. A cytokine release syndrome was observed in 2 of 7 patients treated with a 25 mg flat dose. No tumor response was reported, but the number of patients was very small. Pharmacokinetic analyses revealed that the half-life of the antibody in the serum was surprisingly short (between 4 and 8 h). This could account for the lack of efficacy, as serum concentrations never reached the concentration hypothesized to be required for activity in preclinical studies [84].

#### 3.2.3 Soluble TGF-β receptor traps

Receptor traps are made of soluble (often dimeric) versions of the extracellular domains of cytokine receptors. The traps do not possess kinase activity and they consume the corresponding circulating cytokines. TGF-β traps are made of the extracellular domains of two TGF-βRII chains fused to an antibody Fc fragment. While having a high avidity for TGF-β1 and TGF-β3, the TGF-βR trap only poorly binds TGFβ-2 [85], except when the trap is designed to encompass the TGF-βRIII ectodomain (β-glycan). In murine models of spontaneous or transplantable mammary tumors, the TGF-βRII trap markedly decreased numbers of metastases. This effect appeared to result from decreased motility of tumor cells [86]. The TGF-βRII trap is currently tested in a phase I trial in humans.

Another very interesting trap is M7824, (recently re-named bintrafusp alpha). M7824 is a bifunctional fusion protein, made of a fully human IgG1 monoclonal antibody against PD-L1 (avelumab), fused to the extracellular domain of human TGF-βRII. It functions as a TGF-β1, -β2, -β3 trap and also blocks the PD-1/PD-L1 pathways [87]. Addressing the trap to tissues in which PD-L1 is expressed (such as tumors) is expected to reduce systemic TGF-β inhibition and related toxicities. M7824 was tested *in vivo* in several transplantable mouse tumor models. This is feasible thanks to the cross-reactivity of avelumab on mouse PD-L1. M7824 demonstrated a superior anti-tumor activity than corresponding anti-PD-L1 or TGF-βRII trap monotherapies [87]. The molecule entered a phase I dose escalation trial to evaluate its safety. Nineteen patients with different tumor types received the treatment: 26% of the patients experienced serious immune-related adverse events, and three patients had to stop the treatment. In addition, two patients developed keratoacanthomas, which regressed after the therapy was discontinued. Overall, the safety profile of M7824 is similar to that of anti-PD-1/PD-L1 therapies alone, except for keratoacanthomas, which are observed with drugs targeting TGF-β1. Notwithstanding these toxicities, three patients experienced objective responses. A larger phase I clinical trial was thus initiated with 80 NSCLC patients. 21.3% showed objective responses, compared to the 12% and 18% previously reported for avelumab (anti-PD-L1) or pembrolizumab (anti-PD-1), respectively administered as monotherapies in similar cohorts of patients [88]. The objective response rate of M7824 reaches up to 85.7% (6 of 7 patients) in patients with high tumoral PD-L1 expression (*i.e.*, ≥80% PD-L1+ tumor cells by IHC). Although direct comparison is not possible and patient number is very low, these results are encouraging, as historical responses to anti-PD-1 in PD-L1^high^ NSCLCs range from 29.1 to 43.9%.

Of note, another group also developed similar fusion molecules, in which the extra-cellular domain of TGF-βRII was fused to anti-CTLA-4 (ipilimumab), to anti-PD-L1 (avelumab), or to yet another anti-PD-L1 (atezolizumab) [89]. Although these molecules have not yet been tested in humans, as far as we know, interesting results were observed in NSG mice reconstituted with a human immune system. The authors report anti-tumor efficacy with each of the 3 fusions when they were administered to NSG mice that had been reconstituted with human HLA.A2^+^ BM-derived CD34^+^ cells, then grafted with HLA.A2^+^ human tumors. Each fusion molecule had a superior anti-tumor effect by comparison to the co-administration of the corresponding pair of recombinant proteins. These results suggest that blocking TGF-β activated in the close vicinity of Tregs, thanks to anti-CTLA-4 antibody, or in the close vicinity of other immune cells or tumor cells, thanks to the anti-PD-L1 antibody, is not only sufficient for anti-tumor activity, but also more efficient than systemic blockade of TGF-β.

Nevertheless, for neither of the various TGF-β traps fused to an antibody moiety is there conclusive evidence that addressing the fusion to tumor tissues actually reduces systemic inhibition of TGF-β signals. In addition, traps or anti-TGF-β antibodies target mature cytokines and thus compete with endogenous TGF-βRs for binding. The ubiquitous expression of TGF-βRs and the very short half-life of the mature cytokine once released from LAP, are susceptible to reduce significantly the efficacy of these approaches. To circumvent direct competition between endogenous receptors and inhibitory molecules, several groups developed antibodies that bind latent TGF-β1 and aim at blocking its activation. Anti-LAP and anti-GARP:TGF-β1 antibodies belong to this category of TGF-β inhibitors.

#### 3.2.4 Anti-LAP antibodies

Martin et al. recently developed antibodies that bind the latency lasso of LAP (*i.e.* the latency associated peptide of TGF-β1) [90]. Blocking anti-LAP mAbs reinforce the straightjacket that LAP imposes on mature TGF-β1, and prevent activation of the cytokine. Anti-LAP are expected to block release of mature TGF-β1 from all latent TGF-β1 reservoirs, whichever the large protein to which latent TGF-β1 is bound (LTBPs in the extracellular matrix, GARP or LRRC33 on the surface of cells, or other, as yet unknown, proteins). Martin et al have shown in murine models that blocking anti-LAP antibodies increase the anti-tumor activity of anti-PD-1, and propose to further develop the antibodies for clinical use [90]. Efficacy of anti-LAP antibodies might turn out to be superior to that of antibodies neutralizing mature TGF-β isoforms, because anti-LAP bind latent TGF-β1 prior to activation and release of mature TGF-β1. Thus, anti-LAP do not compete for binding to mature TGF-β1 with ubiquitously expressed, high affinity TGF-β receptors. Thanks to their specificity for TGF-β1, they may display reduced toxicities by comparison to inhibitors of all three TGF-β isoforms, and notably reduced cardio-vascular toxicities thanks to preservation of TGF-β2 activity. However, TGF-β1 exerts dual activities in the context of cancer, which include tumor-suppressive activities on pre-malignant cells and pro-tumoral activities during cancer progression and metastasis (see above). Anti-LAP mAbs are expected to inhibit all TGF-β1 activities, including tumor-suppressive activities, and may thus cause undesired toxicities.

#### 3.2.5 Anti-GARP:TGF-β1 antibodies

**Fig. 6:**
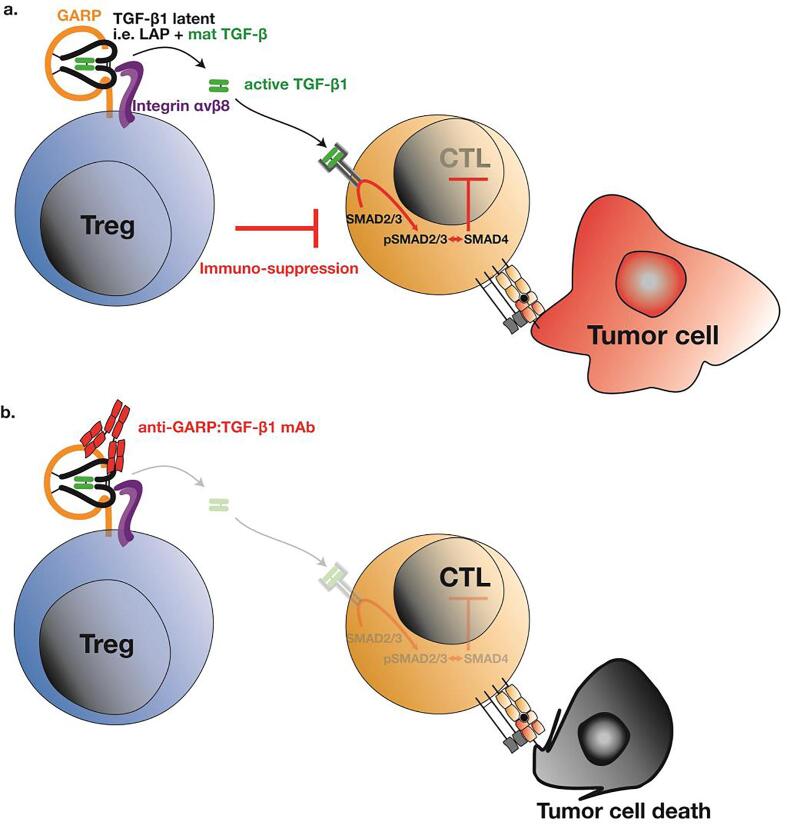
Targeting active TGF-β1 production by Tregs. a Tregs produce active TGF-β1 via GARP and integrin αVβ8. Active TGF-β1 can suppress effector T cells and could contribute to tumor escape. b An anti-GARP:TGF-β1 monoclonal antibody blocks active TGF-β1 production by Tregs. Our hypothesis is that consequently, CTLs would not receive TGF-β1 inhibitory signals and could be more efficient to eliminate tumor cells.

Our lab has developed antibodies that target GARP:(latent)TGF-β1 complexes and prevent TGF-β1 activation on the surface of GARP-expressing cells. GARP:TGF-β1 complexes are expressed by a restricted number of cell types, and even fewer of these activate the cytokine in a GARP-dependent manner. Tregs stimulated *via* their T cell receptor (TCR), B cells stimulated *via* their B cell receptor (BCR), and platelets activated during coagulation *in vitro* were shown to produce active TGF-β1 in a GARP-dependent manner. B cells are not thought to play a major role in anti-tumor immunity. Tregs, in contrast, clearly exert deleterious immune suppression on anti-tumor T cells. Blocking TGF-β1 activation on the surface of stimulated Tregs with anti-GARP:TGF-β1 antibodies could thus represent an interesting approach to block the TGF-β1-mediated immunosuppression by these cells (Fig. 6), without affecting the functions exerted by TGF-β1 activated in a GARP-independent manner by other cells. Our lab previously showed that immunosuppression by human Tregs could be blocked *in vivo* by anti-GARP:TGF-β1 antibodies, in a model of xenogeneic graft-versus-host disease in NSG mice transplanted with human PBMCs [25]. More recently, we reported that anti-GARP:TGF-β1 combined with anti-PD-1 induced immune-mediated rejections of CT26 and MC38 tumors resistant to anti-PD-1 alone [67]. Blocking TGF-β1 activation by GARP-expressing Tregs was sufficient for anti-GARP:TGF-β1 to overcome resistance to anti-PD-1 in these tumor models. Indeed, the anti-tumor activity of combined GARP:TGF-β1/PD-1 blockade: *i)* occurred without Treg depletion, *ii)* was observed using anti-GARP:TGF-β1 incapable of binding Fcg receptors, and *iii)* was lost in MC38 tumor-bearing mice carrying a Treg-specific deletion of the *Garp* gene. In contrast, blocking TGF-β1 activation by GARP-expressing platelets was not required, as anti-tumor activity of combined GARP:TGF- β1/PD-1 blockade was conserved in MC38 tumor-bearing mice carrying a platelet-specific deletion of the *Garp* gene. Thus, in MC38, the predominant source of active TGF-β1 that needs to be blocked by anti-GARP:TGF-β1 to overcome resistance to anti-PD1 are Tregs, but not platelets [67]. Further characterizing its mode of action, we observed that combined GARP:TGF-β1/PD-1 blockade increased the effector functions of anti-tumor CD8 T cells already present within CT26 tumors, without augmenting the immune cell infiltration. Together with our observation that GARP-expressing Tregs are found mostly in human melanoma metastases that are already infiltrated by activated T cells, this led us to suggest testing anti-GARP:TGF-β1 to overcome resistance to PD-1/PD-L1 blockade in patients with inflamed tumors [67]. Anti-GARP:TGF-β1 mAbs are currently tested in a phase I clinical trial in cancer patients (NCT03821935).

## 4 Conclusion

TGF-β1 is a pleiotropic cytokine, the production of which is tightly regulated at multiple levels after translation. Virtually all cells produce the cytokine in a latent form. Depending on the cell type, latent TGF-β1 is produced in association with large proteins that either sequester latent TGF-β1 in the extracellular matrix (for LTBPs), or alternatively present it on the surface of cells such as Tregs (for GARP) or myeloid cells (for LRRC33). This mode of production results in the existence of multiple pools, or reservoirs, of latent TGF-β1 that can be activated through several cell-type specific mechanisms. Only a few cell types activate latent TGF-β1, a process which implies release of the mature TGF-β1 dimer from the LAP pro-peptide. Once activated, mature TGF-β1 can bind ubiquitously expressed high affinity TGF-βRs and engage a signaling cascade that activates or represses expression of TGF-β target genes. Here again, cell-specific and context-specific transcription factors greatly influence the identity of the regulated genes, allowing further tuning of TGF-β1 activity. As a consequence of this complex multi-step regulation, TGF-β1 exerts different activities depending on *i)* the cells that produce it (*i.e.,* the cellular source); and *ii)* the cells that are under its influence in the close vicinity of the source. In the context of cancer, TGF-β1 affects the proliferation and activity of many immune and non-immune cells, including tumor cells themselves. This explains why TGF-β1 is a coveted, but also difficult target for cancer therapy, as many strategies manipulating this pleiotropic cytokine raise concerns regarding safety. Most trials testing strategies to target the active TGF-β isoforms (TGF-β1, -β2 and -β3) turned out to be disappointing. Notwithstanding this, more and more groups propose to develop combinations which include targeting TGF-β signalling along with immune checkpoint blockade, chemotherapy or radiotherapy. This is notably due to observations that TGF-β1 gene signatures are associated with resistance to PD-1/PD-L1 blockade in patients with melanoma or metastatic urothelial cancer [91], [92].

This review emphasizes the multifaceted regulation of TGF-β1 activities and its roles in the context of cancer. It also highlights recent discoveries that demonstrate the feasibility of selectively targeting the TGF-β1 activity emanating from a restricted cellular source with monoclonal antibodies or fusion molecules. Notably, anti-GARP:TGF- β1 monoclonals allow to block TGF-β1 activation by GARP-expressing Tregs and overcome resistance to PD-1/PD-L1 blockade in mouse models of cancer. Clinical trials are ongoing to explore these novel avenues, which bear the potential to increase both the safety and the efficacy of strategies targeting TGF-β signaling for the purpose of cancer immunotherapy.

## CRediT authorship contribution statement

**Grégoire de Streel:** Writing - review & editing. **Sophie Lucas:** Writing - review & editing.

## Declaration of Competing Interest

The authors declare the following financial competing interests: Patents pertaining to anti-GARP:TGF-β1 antibodies discussed in the review article have been filed under the Patent Cooperation Treaty (international application Numbers PCT/EP2014/066650 and PCT/1B2019/053753), with Sophie Lucas and Grégoire de Streel as inventors and UCLouvain as applicant.
